# Exploring the diversity and genetic structure of the U.S. National Cultivated Strawberry Collection

**DOI:** 10.1093/hr/uhac125

**Published:** 2022-05-26

**Authors:** Jason D Zurn, Kim E Hummer, Nahla V Bassil

**Affiliations:** Department of Plant Pathology, Kansas State University, Manhattan, KS, United States of America; USDA-ARS National Clonal Germplasm Repository, Corvallis, OR United States of America; USDA-ARS National Clonal Germplasm Repository, Corvallis, OR United States of America

## Abstract

The cultivated strawberry (*Fragaria* ×*ananassa*) arose through a hybridization of two wild American octoploid strawberry species in a French garden in the 1750s. Since then, breeders have developed improved cultivars adapted to different growing regions. Diverse germplasm is crucial to meet the challenges strawberry breeders will continue to address. The USDA-ARS National Clonal Germplasm Repository (NCGR) in Corvallis, Oregon maintains the U.S. strawberry collection. Recent developments in high-throughput genotyping for strawberry can provide new insights about the diversity and structure of the collection, germplasm management, and future breeding strategies. Genotyping was conducted on 539 *F.* ×*ananassa* accessions using either the iStraw35 or FanaSNP 50 K Axiom array. Data for markers shared by the two arrays were curated for call quality, missing data, and minor allele frequency resulting in 4033 markers for structure assessment, diversity analysis, pedigree confirmation, core collection development, and the identification of haplotypes associated with desirable traits. The *F.* ×*ananassa* collection was equally diverse across the different geographic regions represented. K-means clustering, sNMF, and UPGMA hierarchal clustering revealed seven to nine sub-populations associated with different geographic breeding centers. Two 100 accession core collections were created. Pedigree linkages within the collection were confirmed. Finally, accessions containing disease resistance-associated haplotypes for *FaRCa1*, *FaRCg1*, *FaRMp1*, and *FaRPc2* were identified. These new core collections will allow breeders and researchers to more efficiently utilize the *F.* ×*ananassa* collection. The core collections and other accessions of interest can be requested for research from the USDA-ARS NCGR via the Germplasm Resources Information Network (https://www.ars-grin.gov/).

## Introduction

The cultivated strawberry (*Fragaria* ×*ananassa*) arose through the hybridization of the wild American strawberry species *Fragaria chiloensis* and *Fragaria virginiana* in a French garden in the 1750s (ref. 1). Since that event, the sweet fruit has become popular globally and now encompasses a $15.9 billion global industry [2]. In the United States between 2014 and 2019, an average of 21 474 hectares (53 064 acres) of strawberries were harvested for the fresh and processing markets at an average estimated value of $2.7 billion per year [3]. Regional breeding programs have been established globally to meet the needs of local growers and consumers. These programs focus on enhancing horticultural traits such as fruit sweetness or resistance to endemic diseases.

Diverse germplasm collections are essential when identifying novel traits for future breeding efforts. Incorporation of new germplasm is critical so that cultivars meet the challenges posed by intensifying demand, climate change, and water and land shortages [4]. Approximately, 1700 genebanks have been established worldwide to preserve historic cultivars, landraces, and wild crop relatives. In the United States, the U.S. Department of Agriculture Agricultural Research Service (USDA-ARS) maintains 21 research units tasked with maintaining seed and/or clonally propagated plant germplasm collections [5]. The USDA-ARS National Clonal Germplasm Repository (NCGR) in Corvallis, Oregon, maintains collections of 14 major genera of plants. This includes the *Fragaria* collection, exceeding 1900 accessions from 42 countries. The collection is maintained as containerized clonally propagated plants for each cultivar or selection of the primary gene pool of *F.* ×*ananassa*, and as seedlings or seeds for wild relatives. The *F.* ×*ananassa* cultivars and selections consist of 539 of these accessions.

Over the last few decades, the size of germplasm collections stored in genebanks and the costs associated with maintaining these collections has increased [6, 7]. Moreover, the maintenance of clonally propagated collections, such as the *Fragaria* collection, has additional associated costs not found in seed propagated collections. Breeders, geneticists, and genebank curators often do not have the resources to characterize every accession in a collection genotypically and phenotypically [5–8]. The lack of information due to resource shortfalls is a deterrent when screening germplasm collections for novel phenotypes and can influence genebank management decisions. Core collections are a solution to the challenges surrounding maintaining ever increasing germplasm collections. Core collections are groups of accessions that represent the majority of allelic diversity while having minimal redundancy [9]. Typically, these core collections in genebanks represent approximately 10% of the total collection size, however this concept has been extended toward the development of mini-cores that are approximately 1% of the total collection [9, 10]. Moreover, core and mini-core collections can be designed to reflect different allelic or phenotypic distributions or specific geographic regions, time periods, or breeding goals [5, 7–10]. Regardless of the design goals, core and mini-core collections improve access to germplasm collections and allow collection managers to more efficiently manage a large collection [5].

An initial core subset of 447 *Fragaria* cultivars and world species was identified in the 1980s by the strawberry curator and the Small Fruit Crop Germplasm Committee members to represent maximum genetic diversity. Designation of the core was based on key morphological traits and the locality of the cultivar releases. Molecular information was not available at that time and very little has been done to characterize these accessions genotypically. Moreover, the size of this collection is somewhat large and is not easy to quickly distribute. Recently, strawberry has begun to experience benefits from the genomics revolution. Chromosome-scale genome assemblies have been developed for the diploid *Fragaria vesca* ‘Hawaii-4’ (ref. 11) and *F.* ×*ananassa* ‘Camarosa’ [12] and multiple genome-wide genotyping platforms have been created [13–15]. These new tools have allowed for the dissection of the genetics underlying many important horticultural traits [16–21], disease resistance traits [17, 22–26], as well as the exploration of genetic diversity [27, 28]. Applying molecular information to the *F. ×ananassa* collection can result in core collections that better represent the diversity present in the collection.

Since the original core designation, 160 additional strawberry cultivars were received by the USDA-ARS NCGR and very little has been done to explore the molecular diversity within the U.S. National *F. ×ananassa* collection. As such, we highlight the diversity present in the U.S. National *F.* ×*ananassa* collection, establish core collections based on molecular information, and describe the prevalence of disease resistance and horticultural quality associated haplotypes within the collection to better aid germplasm preservation and breeding efforts.

## Results

### Data Curation

For the accessions genotyped with the Axiom IStraw35 array, 27 968 SNP markers were classified as “No Minor Homozygote” or “Poly High Resolution”. There were 40 424 SNP markers that were classified as “No Minor Homozygote” or “Poly High Resolution” for the accessions genotyped with the Axiom FanaSNP array. After combining the datasets, 4152 markers were identified that were shared between the two Axiom arrays that met the quality classifications. All 539 accessions had less than 15% missing data and were retained. There were 4143 markers that had less than 10% missing data that were retained for analysis. Finally, 110 markers had minor allele frequencies less than 0.05 and were excluded resulting in 4033 markers in the final dataset (Supplementary Data S1).

**Figure 1:**
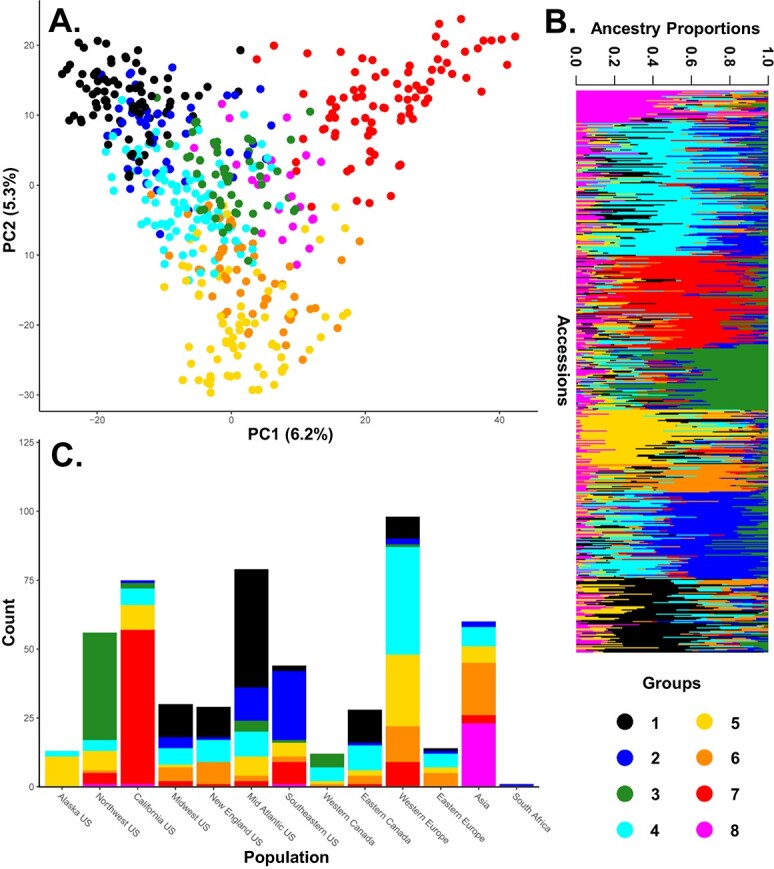
Structure of the U.S. National *F. ×ananassa* collection as determined using (A.) PCA and K-means clustering, (B.) sparse non-negative matrix factorization sorted by ancestry as determined by LEA, and (C.) the distribution of groups assigned via k-means clustering across geographic regions. Colors representing groups have been standardized across the figure.

### Population structure and diversity analysis

**Figure 2:**
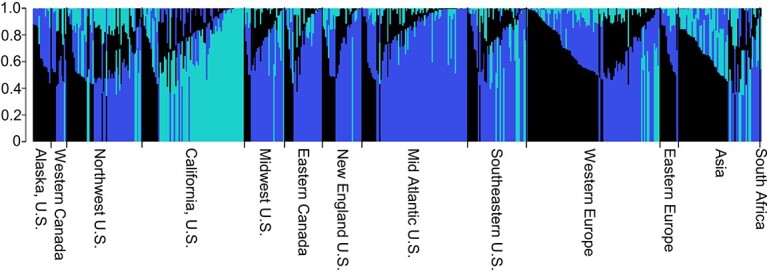
Structure of the U.S. National *F. ×ananassa* Collection as determined using STRUCTURE [37] and STRUCTURE HARVESTOR [38, 39]. Subpopulations were visualized using Structure Plot v2.0 (ref. 40).

When evaluating population structure using PCA, the first two principal components explained 6.2% and 5.3% of the structural variance (Fig. 1A; Supplementary Table S1). Subsequent principal components each explained less than 2.5% of the variance. Principal component one primarily separated Californian germplasm from the other germplasm (Fig. 1A & 1C). The second principal component separated American and European germplasm across a gradient (Fig. 1A & 1C). When applying k-means clustering to the data, the BIC rapidly decreased for the first four groups and continued to decrease reaching a minimum value at eight groups before increasing. Therefore, eight groups were determined to be the optimum number of subpopulations. Conducting population structural analysis using the sNMF algorithm also identified eight clusters (Fig. 1B). A large amount of admixture was observed for each group when looking at the results of the sNMF algorithm. No clear admixture patterns were observed between groups which is consistent with the large amount of historic germplasm sharing between different geographical breeding programs. Accessions grouped similarly when using k-means clustering and sNMF (Table 1). Interestingly, most clustering groups correlated with geographical strata to some degree (Fig. 1C; Table 1; Supplementary Table S1). Group 1 primarily consisted of accessions from the Mid Atlantic U.S., Eastern Canada, the Midwest, and New England; group 2 of accessions from the Southeastern U.S. and Mid Atlantic US; group 3 of accessions from the Northwest U.S. and Western Canada; and group 7 of accessions from California. Groups 4 and 5 were generally associated with European cultivars. Group 8 consisted almost entirely of accessions from Japan. Finally, group 6 was a mix of European and Asian accessions. Hierarchal clustering using UPGMA identified between seven and nine major clades (Supplementary Table S1; Supplementary Fig. S1). Like the results of k-means clustering and sNMF these clades were associated with geographical strata (Table 1). The k-means clustering and sNMF results also correlated with the UPGMA clustering (Table 1). When evaluating population structure using STRUCTURE and STRUCTURE HARVESTER, three subpopulations were identified (Fig. 2). One subpopulation consisted primarily of accessions from California, one that was broadly North American, and the last contained a mix of Asian and European accessions.

**Table 1:** Cramér’s V for the population structure results using sNMF, k-means clustering, UPGMA clustering with seven clades, and UPGMA with nine clades. A Cramér’s V above 0.5 indicates the two methods are highly correlated

	Geographic Origin	sNMF	k-means
sNMF	0.5180		
k-means	0.5334	0.7419	
UPGMA 7 Clades	0.5367	0.6997	0.7358
UPGMA 9 Clades	0.5350	0.7719	0.7834

When looking at the diversity statistics, the richness (eMLG) and evenness were found to be identical between each of the geographic populations (Table 2). The geographic regions were found to be similarly diverse, with Simpson’s index (1 – λ) ranging from 0.92 to 0.99. When expressed as 1 – λ, Simpson’s index is the probability that two individuals randomly selected from a population are genetically different. Nei’s expected heterozygosity ranged from 0.29 to 0.37, which is expected of breeding germplasm and cultivars. Pairwise F_ST_ values for the different geographic regions were very low with values ranging from 0.0018 to 0.1135. The smallest F_ST_ value was observed between the Midwest U.S. and New England U.S. geographic regions and the largest between California, U.S. and Alaska, U.S.

**Table 2:** Comparison the U.S. National *F. ×ananassa* Collection as separated by geographic region of origin. The number of accessions per region (N), richness (eMLG), Simpson’s index (1-λ), evenness, Nei’s expected heterozygosity (H_Exp_), number of accessions included in the type 1 core collection (CC-I), and number of accessions included in the type 2 core collection (CC-X) are shown. The South African region was excluded due to a sample size of one. The accession from South Africa is in the type 2 core collection but not included in the count shown

**Region of Origin**	**N**	**eMLG**	**1-λ**	**Evenness**	**H**_**Exp**_	**CC-I**	**CC-X**
Alaska, U.S.	13	12	0.92	1.00	0.29	2	1
Northwest U.S.	56	12	0.98	1.00	0.34	13	11
California, U.S.	75	12	0.99	1.00	0.37	17	13
Midwest U.S.	30	12	0.97	1.00	0.36	4	8
New England U.S.	29	12	0.97	1.00	0.36	8	7
Mid Atlantic U.S.	79	12	0.98	1.00	0.35	12	7
Southeastern U.S.	44	12	0.98	1.00	0.37	8	9
Western Canada	12	12	0.92	1.00	0.34	3	2
Eastern Canada	28	12	0.96	1.00	0.36	2	6
Western Europe	98	12	0.99	1.00	0.36	19	20
Eastern Europe	14	12	0.93	1.00	0.34	2	2
Asia	60	12	0.98	1.00	0.35	10	13
**All Individuals**	538	12	1.00	1.00	0.37	100	99

### Core collection creation

Type 1 (CC-I) and type 2 (CC-X) core collections were created (Supplementary Table S1). Each of these collections consisted of 100 individuals. The A-NE criterion of the CC-I collection was minimized to a value of 0.122. When random sampling 100 individuals without replacement and calculating the A-NE criterion 1000 times the criterion was 0.151 ± 0.002. The CC-X core collection’s E-NE criterion was maximized to 0.249. The E-NE criterion was 0.186 ± 0.007 when randomly sampling 100 individuals without replacement and calculating the E-NE criterion 1000 times. Both cores are significant (α = 0.05) improvements compared to randomly sampling 100 individuals. Both cores represent accessions originating from each of the major geographic regions (Table 2) and contain individuals representing all groups from the population structure analysis (Supplementary Table S1). When looking at Simpson’s index (1 – λ), evenness, and Nei’s expected heterozygosity, the values were 0.99, 1.00, and 0.37, respectively, for each of the collections. The high Simpson’s index and evenness reflect the diversity captured by each of the core collections. Moreover, the diversity statistics are the same as those reported for the whole *F.* ×*ananassa* collection (Table 2), further demonstrating that the diversity of the collection is reflected in the created core collections. Of the 539 accessions analyzed, 172 were represented in at least one of the cores. A set of 28 individuals can be found in both cores, of which 13 are the cultivars used as “seeds” for the core construction. The 15 found in both cores that were not preselected “seeds” are: ‘Aiberry’ (PI 641175), CA 39.117–4 (PI 551672), CA 59.39–1 Rockhill 2^nd^ BC (PI 551675), ‘Columbia’ (PI 551760), *F.* ×*ananassa* 86.51.10 (PI 616615), ‘Jewel’ (PI 551927), ‘Lambada’ (PI 617021), ‘Marys Peak’ (PI 682649), ‘Northland’ (PI 551592), ORUS 2427–1 (PI 670236), ‘Redcoat’ (PI 551596), ‘Selva’ (PI 551814), ‘Stoplight’ (PI 551808), ‘Touhoku 10’ (PI 616630), and ‘Vesper’ (PI 551602).

### Pedigree confirmation

When looking at IBS calculations, 64 sets of individuals were considered to be synonyms (Supplementary Table S2). Some of these accessions are expected to be identical as they were either received from two different sources, meristem cultures of the same accession, or sports of an accession. Eleven sets of plants that met one of these classifications existed within the dataset. Two different clones (CFRA 310.002 and CFRA 310.003) of the accession PI 551674 (CA 51S1–1 Sequoia parent) were sampled and found to be identical. The accessions PI 616860 (‘Venta’) and PI 616861 (‘Venta’) received as individual plants at the same time were found to be identical. Additionally, PI 551716 (CA 64.28–18) and PI 551717 (CA 64.28–18 8x) were found to be identical. PI 551411 (‘Tamella’) and PI 666638 (‘Tamella’ – Netherlands) that were received from different sources were identical. This was also true of PI 551548 (‘Tioga’) and PI 551667 (‘Tioga’), which were also received from different sources. PI 552235 and PI 552236 were different meristem cultures of the same accession, ORUS 3727, and had identical fingerprints, as expected. PI 551544 (‘Early Midway’) was suspected to be a sport of PI 551538 (‘Midway’) and was found to be a true sport. Finally, PI 664446 (‘Sparkle Supreme’) is a putative sport of PI 551559 (‘Sparkle’) and was found to be a true sport. The accessions PI 551581 (‘Korona’) and PI 666636 (‘Korona’ – Netherlands) were found to be different, with PI 666636 representing the genotype used as a parent for the van Dijk *et al.* (2014) ‘Holiday’ × ‘Korona’ genetic map. The accessions PI 551630 (‘Aberdeen’ – Netherlands) and PI 666634 (‘Aberdeen’ – New Jersey) were found to be different. Four clones of ‘Marshall’ (PI 231090, PI 551842, PI 684677, and PI 691745) had different genotypes. Individuals that were not expected to be synonyms are likely clones that were mistakenly mislabeled when acquired or during routine repropagation or are in pots that are physically nearby in the USDA-ARS NCGR screen house. For the accessions that are nearby in the USDA-ARS NCGR screenhouse, it is likely a runner from one pot established a plantlet in an adjacent pot and displaced the original clonal accession.

When evaluating the COLONY output, 308 accessions could be evaluated for pedigree links by either having a putative parent or offspring within the collection (Supplemental Table S3; Supplemental Fig. S2). Pedigree links were established for 241 accessions and of the 309 accessions that could be assessed, 78.0% of them appear to be true-to-type (TTT). For these accessions the pedigree links matched the previously reported pedigrees, confirming they are TTT. Notably, PI 666634 (‘Aberdeen’ – Netherlands) and PI 691745 (‘Marshall’) were able to be identified as TTT ‘Aberdeen’ and ‘Marshall’, respectively. Many of the hypothetical parents of the accessions in the collection are not present and pedigree links or trueness-to-type could not be established. Pedigree linkage was able to identify the TTT accession for 29 of the of the 58 sets of accessions that were synonyms and not expected to be accessions received from two different sources, meristem cultures of the same accession, or sports of an accession. Finally, six individuals were identified that appear to be TTT based on pedigree linkage but will require further investigation. These six accessions are ‘Badgerglow’ (PI 551636), ‘Cesena’ (PI 551754), ‘Dana’ (PI 551756), ORUS 4357 ORUSM 202 (PI 551856), ‘Redcrest’ (PI 551859), and MDUS 4355 (PI 551934). ‘Badgerglow’ is pedigree linked to its putative offspring ‘Gilbert’ (PI 551587), but not to its putative TTT parents ‘Sparkle’ (PI 551559) and ‘Stelemaster’ (PI 551614). It is possible that the pedigree of ‘Badgerglow’ was miss-recorded. The accessions of ‘Cesena’ and ‘Dana’ within the collection are synonymous based on IBS. ‘Tago’ (PI 551599) is the putative parent of ‘Cesena’ and can be pedigree linked. ‘Linda’ (PI 616618) is a putative offspring of ‘Dana’ and could be linked based on pedigree. ORUS 4357 ORUSM 202 and ‘Redcrest’ are synonyms based on IBS and are full siblings that are pedigree linked to their reported parents ‘Linn’ (PI 551500) and ‘Totem’ (PI 551501). Additionally, pedigree linkage was established between ‘Redcrest’ and its putative offspring ORUS 1267–236 (PI 651548). It is possible that the markers used are not able to differentiate ‘Cesena’ and ‘Dana’ and ORUS 4357 ORUSM 202 and ‘Redcrest’. Alternatively, there may have been a propagation error. MDUS 4355 was pedigree linked to ‘Mohawk’ (PI 616598) which has a putative pedigree of MDUS 4587 (PI 551936) and TTT ‘Earliglow’ (PI 551394). MDUS 4587 is synonymous with the TTT MDUS 4645 (PI 551939). A propagation error may have occurred where MDUS 4355 was displaced by MDUS 4587 and MDUS 4587 was then subsequently displaced by MDUS 4645. To resolve the problems surrounding these individuals, additional genotyping and comparisons with these accessions maintained at other institutions or genebanks will be needed.

### Trait associated haplotype prevalence

**Figure 3:**
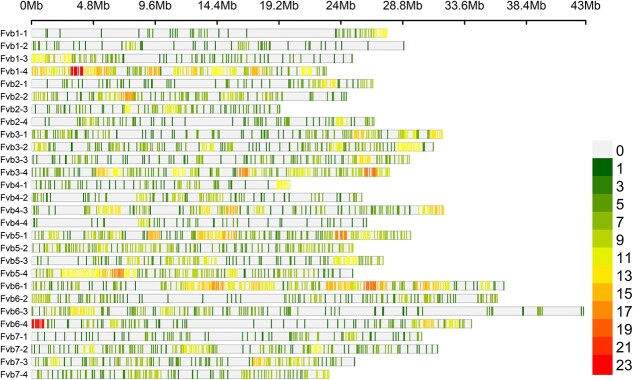
Distribution of the filtered markers across the *F. ×ananassa* ‘Camarosa’ v1 genome [12].

Only 3943 markers of the 4033 markers mapped uniquely to the *F.* ×*ananassa* ‘Camarosa’ v. 1.0 assembly (Fig. 3; ref. 12). The number of markers mapping to chromosome Fvb1–1 was low relative to the remaining chromosome 1 homoeologs. However, markers were well distributed across the length of each of the chromosomes. Short-range LD decayed to an *r*^2^ of 0.20 at 856 kb. As such, a ± 856 kb window around markers associated with disease resistance or flowering traits was used to describe haplotypic regions.

Unfortunately, marker density was too low in the *FaPFRU*, *Fw1*, and *FaRMp2* regions to identify haplotypes associated with the traits of interest. Resistance haplotypes were identified for *FaRCa1, FaRCg1*, *FaRMp1,* and *FaRPc2* (Table 3; Supplementary Tables S4-S7). *FaRPc2* and *FaRMp1* are known to have multiple haplotypes associated with disease resistance [23, 24]. The *FaRPc2 r*esistance haplotypes are known as *FaRPc2* H2 and *FaRPc2* H3 (ref. 23). Both haplotypes were identified in the collection. *FaRMp1* is known to have three resistance haplotypes, *FaRMp1* H2, *FaRMp1* H3, and *FaRMp1* H4 (ref. 24). Only *FaRMp1* H3 could be identified in the collection. The prevalence of resistance haplotypes for *FaRPc2, FaRCa1, FaRCg1,* and *FaRMp1* was quite high within the collection (Table 3; Supplementary Tables S4-S7). *FaRCa1* was found in 253 accessions, *FaRCg1* in 78 accessions, *FaRMp1* H3 in 205 accessions, *FaRpc2* H2 in 53 accessions, and *FaRPc2* H3 in 307 accessions. These haplotypes could be found globally and most geographic regions had one adapted cultivar containing these resistant haplotypes. Additionally, these haplotypes are all represented within each of the developed core collections. The accessions PI 551484 (‘Fletcher’), PI 616579 (US-159), and PI 664345 (‘L’Amour’) are of note as they have multiple resistance associated haplotypes: *FaRCa1*, *FaRCg1*, *FaRMp1* H3, and *FaRPc2* H3 (Supplementary Table S3-S7).

**Table 3:** Distribution of disease resistance haplotypes across geographic regions and within the core collections

	**N**	***FaRCa1***	***FaRCg1***	***FaRMp1 H3***	***FaRPc2 H2***	***FaRPc2 H3***
Alaska, U.S.	13	10	0	3	0	5
Northwest U.S.	56	28	5	18	6	40
California, U.S.	75	36	2	28	6	57
Midwest U.S.	30	13	2	14	0	16
New England U.S.	29	13	10	14	2	17
Mid Atlantic U.S.	79	37	19	37	6	44
Southeastern U.S.	44	22	11	13	4	26
Western Canada	12	5	0	5	0	6
Eastern Canada	28	11	10	9	1	16
Western Europe	98	42	13	41	13	40
Eastern Europe	14	13	0	8	5	6
Asia	60	22	6	14	10	34
South Africa	1	1	0	1	0	0
**All Individuals**	539	253	78	205	53	307
**CC-X Core**	100	61	14	36	12	57
**CC-I Core**	100	41	15	40	9	63

## Discussion

The cultivated strawberry has only recently begun to take advantage of the genomics revolution. Due to new tools, questions surrounding breeding, evolution, and germplasm conservation can be answered in much greater detail than previous examinations. The U.S. National *Fragaria* Collection has long operated without a deep understanding of the relatedness and molecular diversity within the collection. The collection was assumed to be diverse based on the geographic origins of much of the accessions; however, the cultivated strawberry has been considered to have a narrow genetic diversity based on antidotal evidence of a limited number of founding cultivars that were globally shared [1] and genomic analyses of *F. ×ananassa* diversity has both supported and refuted this narrative [27, 28, 55]. As such, a genomic assessment of the collection was critical to facilitate future germplasm management efforts.

### Diversity analysis of the U.S. *F. ×ananassa* collection

An assessment of diversity by geographic origins of the cultivars revealed that diversity within the *F. ×ananassa* is well represented globally. Allelic evenness, richness, and diversity as assessed by Simpson’s index was similar across each region. These metrics should be regarded with some reservations. SNP chips are known to be greatly impacted by ascertainment bias and the bi-allelic nature of the probes will hide diversity that would be observed using methods not restricted to two alleles and allow for the inclusion of rare alleles, such as simple sequence repeat (SSR) markers and genotype-by-sequencing [56–58]. The markers used in the present study were initially identified using a limited set of germplasm [13, 14] and then selected for inclusion on the FanaSNP array based on performance in global germplasm panel that was biased towards California germplasm [14]. Despite these limitations, the data set is sufficient for population structure analysis, core collection creation, and haplotype identification [56–58].

### Population structure of the U.S. *F. ×ananassa* collection

Pairwise F_ST_ between the geographic regions was very low, aligning with narratives of global germplasm sharing within *F. × ananassa* [1, 27, 28, 55]. When looking at the structure of the population, the first two principal components explained very little of the population structure and a large degree of admixture was observed when considering the sNMF algorithm. Again, this is in line with the narrative of global sharing of ancestral *F. ×ananassa* germplasm. K-means clustering and structure analysis using the sNMF algorithm separated the *F. ×ananassa* collection into eight groups that corresponded primarily with geographic regions or prominent breeding programs. Unfortunately, *F. ×ananassa* accessions from South America or Australia are not present in the collection and these regions may be targets for future germplasm acquisition. It remains to be seen if cultivars from these regions would group distinctly if incorporated into the present data set.

When analyzing the population structure using STRUCTURE and STRUCTURE HARVESTER only three subpopulations were identified (Fig. 2). One cluster consisted primarily of Californian germplasm with the remaining two clusters consisted of germplasm originating from other breeding programs. This result is reflected in the shape of the PCA with Californian germplasm and the other germplasm being explained by the first principal component and the other two subpopulations being separated along the second principal component (Fig 1A. & Fig 1C.). The results using STRUCTURE and STRUCTURE HARVESTER are also consistent with the findings of Hardigan et al. [27]. Hardigan *et al.* [27] observed six subpopulations when conducting structure analysis. Two of these groups were attributed to *F. virginiana* and *F. chiloensis* germplasm. The remaining four groups consisted of post-1990 University of California germplasm, germplasm from the University of Florida, and two admix groups of North America and Europe. Only the Florida and California breeding programs separated uniquely from the remaining *F. ×ananassa* germplasm and were supported by F_ST_ values above 0.13. An F_ST_ value of 0.13 is low and still supports a large amount of admixture despite these populations differentiating from the “cosmopolitan” population. In the present study, the three subpopulations identified by STRUCTURE and STRUCTURE HARVESTER correspond to the two admix North American and European subpopulations from Hardigan *et al* [27]. The separation of California germplasm from global temperate germplasm was also observed in prior research [29]. This is unsurprising as regional breeding goals are expected to change as new challenges arise and would result in population structuring.

Determining population structure can be difficult in the absence of features that allow for population stratification, such as geographic barriers or biological factors that impact gene flow. Multiple methods have been developed to assess population structure each with their own advantages and disadvantages. These approaches can lead to different but equally valid interpretations of population structure. The PCA and sNMF approaches are model free methods for determining population structure while STRUCTURE is a Bayesian modeling approach that has prior assumptions of the data [35, 37]. STRUCTURE assumes the absence of genetic drift, ancestral populations are in Hardy–Weinberg and linkage equilibrium, and that sampling is even among populations [35, 37, 59–61]. The data used in the present study violates some of the assumptions used in STRUCTRUE’s Bayesian model. In particular, sampling was uneven with the majority of the collection containing accessions from North American breeding programs and the USDA-ARS Corvallis, OR and University of California – Davis breeding programs being over represented. Moreover, Hardy–Weinberg equilibrium cannot be assumed. When individuals do not belong to distinct Hardy–Weinberg populations, STRUCTURE can cluster individuals in unpredictable ways [60]. This has typically been observed in situations where individuals mate preferentially with neighboring individuals [62]. In breeding programs, non-random mating would be expected based on parental selection by the breeder. In the presence of unbalanced designs, STRUCTURE and the Evanno method [38, 39] are conservative and tend to underestimate the number of subpopulations and will merge subpopulations represented by smaller numbers [60, 61]. The conservative nature of this approach may help identify relationships between distinct subpopulations.

The lack of distinctly European or North American subpopulations in Hardigan *et al.* [27] and the present study when evaluating the populations using STRUCTURE and the Evanno method may be due to the inclusion of unbalanced subpopulation, violations of Hardy–Weinberg equilibrium, or the conservative nature of the analysis method used. Regardless of the reason, the current work demonstrates the importance of using multiple methods when evaluating population structure. The U.S. National *F. ×ananassa* Collection contains many of the cultivars in the “cosmopolitan” population in Hardigan *et al.* [27] and consists primarily of older cultivars and breeding selections. Moreover, University of Florida breeding program is severely underrepresented within the NCGR collection, despite its importance within the U.S. It is also possible the structural differences identified using k-means clustering, sNMF, and UPGMA hierarchal clustering in the current work may have been masked by the more structured wild and contemporary Florida and California germplasm included in Hardigan *et al.* [27]. Moreover, breeders have recently begun to explore wild *F. virginiana* and *F. chiloensis* germplasm and their natural hybrids for novel traits [24, 29, 63–65]. This would suggest that diversity within the *F. ×ananassa* Collection may be improved by incorporating more contemporary cultivars that have wild origins.

### Core collection creation

Core collections have proven to be a useful tool to quickly screen germplasm collections for desirable traits or identify QTLs via association mapping for many plant species [4, 5, 8]. These collections have often been created using genome-wide information. Cost-effective, genome-wide genotyping platforms have only recently become available for strawberry. As such, developing a core collection that captures the genotypic diversity within strawberry collections was not possible. Prior efforts consisted of establishing strawberry core collections based primarily on geographic origins, limited trait data, and plant morphology. As a result, a large core consisting of 321 *F. ×ananassa* accessions was constructed. Due to its large size, the original core collection likely represented much of the diversity within the collection, albeit inefficiently. Moreover, the original core collection was difficult to easily distribute to researchers due to its large size. There are many ways to develop core collections based on the goals of the research being conducted [7]. Ideally, a “good” core collection should be representative of the whole collection with respect to taxonomic classification and geographic origin, will not have redundant accessions, and be of a size that can be easily managed and distributed [7, 9]. Distance-based criterion, such as the A-NE and E-NE criterion, have been shown to result in CC-I and CC-X collections that are more representative than arbitrarily picking collection members [7]. The two core collections created in the present study represent the diversity of the collection with a much smaller number of individuals. In many ways these core collections fit the definition of a “good” core collection. The *F. ×ananassa* accessions within these new core collections represent all of the geographic origins of cultivars within the collection (Table 2), there are no redundant accessions in these collections, and the sizes of these collections are more reasonable for maintenance and distribution. Moreover, both of these collections were created using distance-based criterion. These cores will be easier to distribute to interested researchers and breeders than the prior core collection and are a much-improved resource for the strawberry research and breeding community due to their associated genotypic data.

### Pedigree confirmation within the U.S. *F. ×ananassa* collection

Pedigree links could be established for about half of the U.S. National *F. ×ananassa* Collection. This is in part due to the historic nature of the collection. Many of the accessions are older cultivars and their parents have not been conserved or are unknown. As such, until putative parents or offspring are added to the collection or accessions from the U.S. National *F. × ananassa* Collection are compared to putative parents or offspring accessions in other germplasm collections, some identities will remain uncertain. Additionally, the repropagation of these cultivars over time has likely led to a few propagation errors, resulting in different accessions being accidently renamed or the accidental retention of offspring rather the original clone. This is a persistent problem in clonally propagated crops and DNA fingerprinting has been used as a way to validate true-to-type identities [28, 58, 66, 67]. Despite this problem, numerous heritage cultivars were identified that are likely true-to-type. A total of 15 accessions that were developed prior to 1945 are likely true-to-type. The oldest of these was ‘Jucunda’ (PI 551623) developed in 1859 (Supplementary Table S1).

### Identification of trait associated haplotypes

The identification of trait associated haplotypes is dependent on marker density and LD decay within a population. The LD decay to an *r*^2^ of 0.2 was 856 kb in the U.S. National *F. × ananassa* Collection. Hardigan *et al.* [27] observed decay at 120 bp in the “cosmopolitan” *F. × ananassa* population and 400 kb in Californian *F. ×ananassa*. The University of Florida strawberry breeding program observed decay to *r*^2^ = 0.2 at 3.5 Mb to 4.2 Mb [68]. The higher values observed by Osorio *et al.* [68] was likely due to the amount of relatedness and selection within the breeding populations [14, 27, 69, 70]. The estimated LD decay in the present study is in range for *F. ×ananassa*. However, LD decay may be reduced due to the lack of rare alleles in the data through the use of an optimized set of bi-allelic markers. Conversely, the lower density of markers used compared to those used in Hardigan *et al.* [27] may have also caused the appearance of reduced LD decay. This lower marker density became problematic when trying to identify haplotypes within the *FaPFRU*, *Fw1*, and *FaRMp2* QTL regions. Marker density was sufficiently high in the regions for the disease resistance QTLs *FaRCa1*, *FaRCg1*, *FaRMp1*, and *FaRPc2*. Resistance associated haplotypes for *FaRCa1*, *FaRCg1*, *FaRMp1*, and *FaRPc2* were globally distributed (Table 3; Supplementary Tables S4-S7). Anthracnose fruit rot, Colletotrichum crown rot, charcoal rot, and Phytophthora crown rot are challenges for many breeding programs globally and it is not unexpected that breeders selected for resistance to these diseases [22–24, 26]. In wild germplasm the prevalence of these haplotypes is likely lower. Few lines contained *FaRCg1* compared to the other resistance genes (Table 3; Supplementary Tables S4-S7). This suggests Colletotrichum crown rot has not been a large breeding focus historically or other resistance genes that have yet to be identified have been primarily used to manage the disease. Interestingly, *FaRPc2* H2 was much less prevalent than *FaRPc2* H3. *FaRPc2* H3 provides more disease resistance than *FaRPc2* H2 and may have been selected more intensely than *FaRPc2* H2 (ref. 23). Conversely, *FaRPc2* H3 may provide resistance to a broader spectrum of *Phytophthora cactorum* races than *FaRPc2* H2 resulting in its more frequent use globally. Additional research will be needed to validate the efficacy of *FaRPc2* H3 against a broader spectrum of *P. cactorum* races. Moreover, few of the accessions have been assessed for disease resistance and research is needed to validate the accuracy of these haplotype predictions.

## Conclusions

Germplasm collections provide a wealth of resources for breeders to develop the next great cultivars. The core collections developed for the U.S. National *F. ×ananassa* Collection will improve the collection’s use and allow for the development of the next great strawberry cultivars. Next research steps include further characterization of the collection both phenotypically and genotypically and the validation of shared accession identity across international germplasm collections. Moreover, the core collections created in the present manuscript will be useful tools for the identification of new genes and the validation of DNA-informed breeding tools. Researchers and breeders interested in evaluating the new core collections can request them for research from the USDA-ARS NCGR via the Germplasm Resources Information Network (https://www.ars-grin.gov/).

## Materials and methods

### Germplasm, DNA extraction, genotyping, and data curation

The *F. ×ananassa* accessions in the USDA-ARS *Fragaria* collection (Supplementary Tables S1) were evaluated. Hardigan *et al.* [29] genotyped 364 accessions using the Axiom IStraw35 array (Thermo Fisher Scientific, Waltham, MA U.S.A.; ref. 15). Young leaf tissue was collected for the 175 accessions that were not genotyped by Hardigan *et al.* [29]. For these 175 accessions, DNA was extracted [30] from 30–50 mg of leaf tissue with the Omega E-Z 96 Plant DNA Kit (OMEGA Bio-Tek Inc., Norcross, GA, U.S.A.) and quantified using the Quant-iT PicoGreen dsDNA Assay Kit (Life Technologies Inc., Carlsbad, CA, U.S.A.). DNA for the 175 accessions were submitted to Thermo Fisher Scientific for genotyping with the FanaSNP 50 K Axiom array [14]. The genotypic data for the 175 accessions were scored using the Axiom™ Analysis Suite (v 4.0; Thermo Fisher Scientific, Inc.) following the best practices described in the software documentation. The R package “SNPolisher” v 1.5.2 was used to assign the markers into one of six quality classes according to their clustering performance. Markers that were assigned into the “No Minor Homozygote” (two defined clusters) or “Poly High Resolution” (three defined clusters) classifications were considered for analysis.

Markers shared by the IStraw35 and FanaSNP arrays were combined into a single data set. Next, accessions with more than 15% missing data were filtered out. Markers with more than 10% missing data points across all remaining accessions were removed. Finally, minor allele frequencies for the remaining markers were calculated and markers with a minor allele frequency of less than 5% were removed. The curated data set was then used in analysis.

### Population structure and diversity analysis

All analyses of diversity and structure were conducted using R v 4.0.3 (ref. 31). Accessions were stratified into 13 geographical regions based on their passport information on GRIN-Global. The regions within North America included: Alaska, U.S., Northwest U.S. (Idaho, Oregon, Washington, and Wyoming), California, U.S., Midwest U.S. (Illinois, Indiana, Iowa, Michigan, Minnesota, Missouri, and Wisconsin), Southeastern U.S. (Arkansas, Florida, Louisiana, Mississippi, North Carolina, Tennessee, Texas, and South Carolina), Mid-Atlantic U.S. (Maryland and Delaware), New England U.S. (Connecticut, Maine, Massachusetts, New Hampshire, New Jersey, and New York), Western Canada (British Columbia and Alberta), and Eastern Canada (Ontario and Nova Scotia). Europe was divided into Western Europe (Belgium, Denmark, France, Germany, Italy, Ireland, the Netherlands, Norway, Sweden, and the United Kingdom) and Eastern Europe (Belarus, Lithuania, Poland, and Western Russia). The Asia geographic region included accessions from Eastern Russia, Japan, and China. Finally, a single accession that originated from South Africa made up the final geographic region.

Four methods were used to evaluate population structure. These methods included principal component analysis (PCA) and k-means clustering, sparse non-negative matrix factorization (sNMF), STRUCTURE and STRUCTURE HARVESTER, and unweighted pair group method with arithmetic mean (UPGMA) hierarchal clustering. Principal component analysis followed by k-means clustering was implemented using adegenet v. 2.1.3 (refs. 32 & 33). When conducting k-means clustering the maximum number of principal components were retained and the optimum number of clusters was selected using the minimum Bayesian information criterion (BIC). The sNMF algorithm as implemented in the R package LEA [34, 35] was used to evaluate population structure and admixture between populations. The number of k subpopulations evaluated ranged from 2 to 14 and each analysis was repeated 10 times. The elbow method was used to identify k clusters for the sNMF algorithm. Sample orders were calculated using CLUMPP v. 1.1.2 (ref. 36) and results were visualized using the barchart function from LEA [34]. STRUCTURE v. 2.3.4 (ref. 37) was also used evaluate population structure and admixture between populations. STRUCTURE was set to run from 2 to 14 subpopulations with 25 000 burn-in steps and 50 000 Markov-Chain Monte Carlo (MCMC) steps. Ten replications were performed per k subpopulation. All remaining parameters were set to default. The optimal number of k subpopulations for the STRUCTURE results was identified using STRUCTURE HARVESTER v. 0.6.94 (refs. 38 & 39). Sample orders were calculated using CLUMPP v. 1.1.2 (ref. 36) and results were visualized using Structure Plot v2.0 (ref. 40). Finally, UPGMA hierarchal clustering was performed on a distance matrix constructed using Prevosti’s absolute genetic distance [41] using the R package poppr v 2.8.7 (refs. 42 & 43).

The geographic sub-populations, except for the South African accession, were evaluated for population richness, intra group diversity, expected heterozygosity, and evenness. Intra group diversity was evaluated using Simpson’s index [44] and expected heterozygosity was evaluated using Nei’s expected heterozygosity [45]. Richness, Simpson’s index, Nei’s expected heterozygosity, and evenness were calculated using the R package poppr v 2.8.7 (refs. 42 & 43). The pairwise fixation index (F_ST_) was also calculated for each geographic sub-population, excluding South Africa, using hierfstat v 0.5–7 (ref. 46) to assess the amount of interbreeding/sharing of germplasm between breeding programs in these regions.

### Core collection creation

Two 100 individual core collections were created using the R package corehunter v. 3.2.1 (ref. 6). The first core collection was a type 1 core collection (also known as a CC-I collection) designed to evenly represent the diversity of the collection. The second was a type 2 core collection (also known as a CC-X collection) designed to represent the extremes of the entire collection. The type 1 collection used the average distance between each accession and the nearest entry (A-NE) criterion and works to minimize this value [47]. Minimizing the A-NE criterion causes a type 1 core collection to evenly represent the genetic diversity found in a larger germplasm collection. The type 2 collection used the average distance between each entry and the nearest neighboring entry (E-NE) criterion and works to maximize this value [7]. By maximizing the E-NE criterion, samples with the greatest genetic distance from one another are added to the type 2 core collection during creation. For each collection, a set of 13 accessions was pre-selected as “seeds”. These individuals were selected based on their geographical origin and because they are positive controls for various DNA tests, were sequenced or a parent of a major mapping population, or have been known to be notable cultivars from their geographic region [12, 48, 49]. These 13 accessions were as follows: ‘Camarosa’ (PI 670238), ‘Charm’ (PI 664911), ‘Deutsch Evern’ (PI 551626), ‘Holiday’ (PI 551653), ‘Korona’ – Netherlands (PI 666636), ‘Mara des Bois’ (PI 687353), ‘Ooishi shikinari 2’ (PI 641185), ‘Senga Sengana’ (PI 264680), ‘Strawberry Festival’ (PI 664337), ‘Tochiotome’ (PI 617008), ‘Totem’ (PI 551501), ‘Tribute’ (PI 551953), and US 4809 (PI 637938). Prevosti’s absolute genetic distance was used in construction of each core collection [41]. The corehunter package was run 2000 times when constructing each core collection, retaining the core collection with minimum A-NE or maximum E-NE criterion depending on the collection type, due to the stochastic algorithms used in the package. Intra group diversity, expected heterozygosity, and evenness were assessed using the previously mentioned diversity statistics to ensure the diversity of the whole *F.* ×*ananassa* collection is reflected in each of the created cores.

### Pedigree confirmation

Percent identity by state (IBS) was calculated between each pair of individuals for all individuals. Individuals with greater than or equal to 98% were considered to be synonyms. The software COLONY v 2.0.6.6 (ref. 50) was used for parentage inference. The parameters polygamy for both males and female, inbreeding mating, without clones, monoecious, and diploid were used to describe hybridization within strawberry. The full-likelihood estimates algorithm with precision set to high was used. All remaining parameters were set to the default. Potential parents with a pairwise likelihood under 90% were excluded as parental candidates unless a full-likelihood estimate was provided.

### Trait associated haplotype prevalence

For haplotype identification, markers from the curated dataset that had been mapped to the *F.* ×*ananassa* “Camarosa” v. 1.0 assembly were used [14]. Data were imputed and phased using Beagle v 5.2 (refs. 51 & 52). Pairwise linkage disequilibrium (LD) was calculated using VCFtools v 0.1.16 (ref. 53) to assess LD decay. Haploblocks for each region of interest were defined as N nucleotides proximally and distally from markers associated with each gene or QTL, where N is the genome-wide distance required to reach an r [2] of 0.20 when estimating LD. The genetic regions for the remontancy gene *FaPFRU* [20] and disease resistance genes *FaRCa1* (anthracnose fruit rot; ref. 26), *FaRCg1* (Colletotrichum crown rot; ref. 22), *FaRMp1* (charcoal rot; ref. 24), *FaRMp2* (charcoal rot; ref. 24), *FaRPc2* (Phytophthora crown rot; ref. 23), and *Fw1* (Fusarium wilt; ref. 25) were investigated. Haplotypes associated with perpetual flowering and disease resistance were identified using previously reported favorable alleles in known positive accessions within the collection. The prevalence of these haplotypes within the collection and their geographical distributions were assessed. Haplotypes with identical sequences were arbitrarily named except for those that have been previously identified. Previously identified haplotypes were named using the gene name followed by any signifying haplotype in previous research.

## Supplementary Material

Web_Material_uhac125

## Data Availability

Data used in analysis is provided in Supplementary Data S1. Core collections and *F. ×ananassa* accessions maintained by the USDA-ARS are available for order though Germplasm Resources Information Network (https://www.ars-grin.gov/).
