# Supplementary material for: Exploring the diversity and genetic structure of the U.S. National Cultivated Strawberry Collection
Source: Hortic Res. 2022 May 26;9:uhac125. doi: 10.1093/hr/uhac125 (PMC9343918; doi:10.1093/hr/uhac125)

Supplemental Figure S1. Phylogenetic tree of the U.S. National F. × ananassa collection. Seven to nine major clades were identified (inner highlighting; dashed line). Geographic origins are displayed as colored circles at the tip points (population legend). The outer tracks display clustering information (cluster legend) for the collection using K-means clustering (8 subpopulations; inner track), sNMF (8 subpopulations; middle track), and STRUCTURE (3 subpopulations; outer track). Plotting was conducted using GGTREE v 2.4.2 (ref. 70).

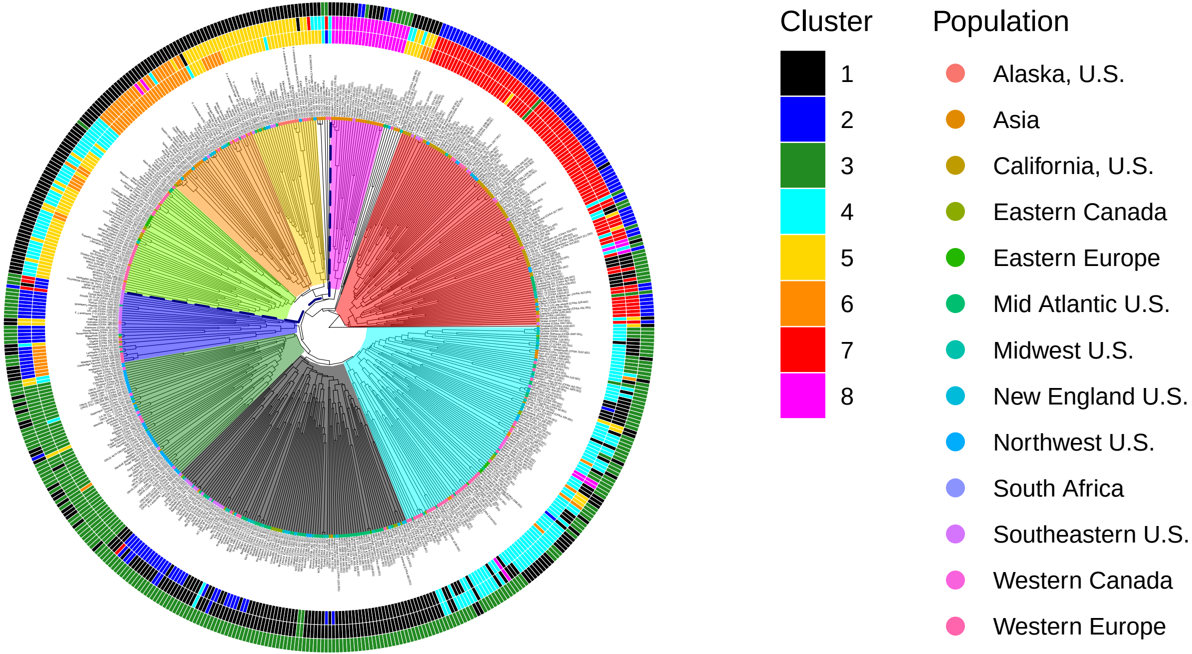

Supplement: Web_Material_uhac125 [file web_material_uhac125.zip › Supplementary_Fig_S1.pdf]
