# Supplementary material for: Exploring the diversity and genetic structure of the U.S. National Cultivated Strawberry Collection
Source: Hortic Res. 2022 May 26;9:uhac125. doi: 10.1093/hr/uhac125 (PMC9343918; doi:10.1093/hr/uhac125)

Supplemental Figure S2. Pedigree links confirmed within the U.S. National F. ×ananassa collection. Figure was produced using Helium v. 1.19.09.03 (ref. 71).

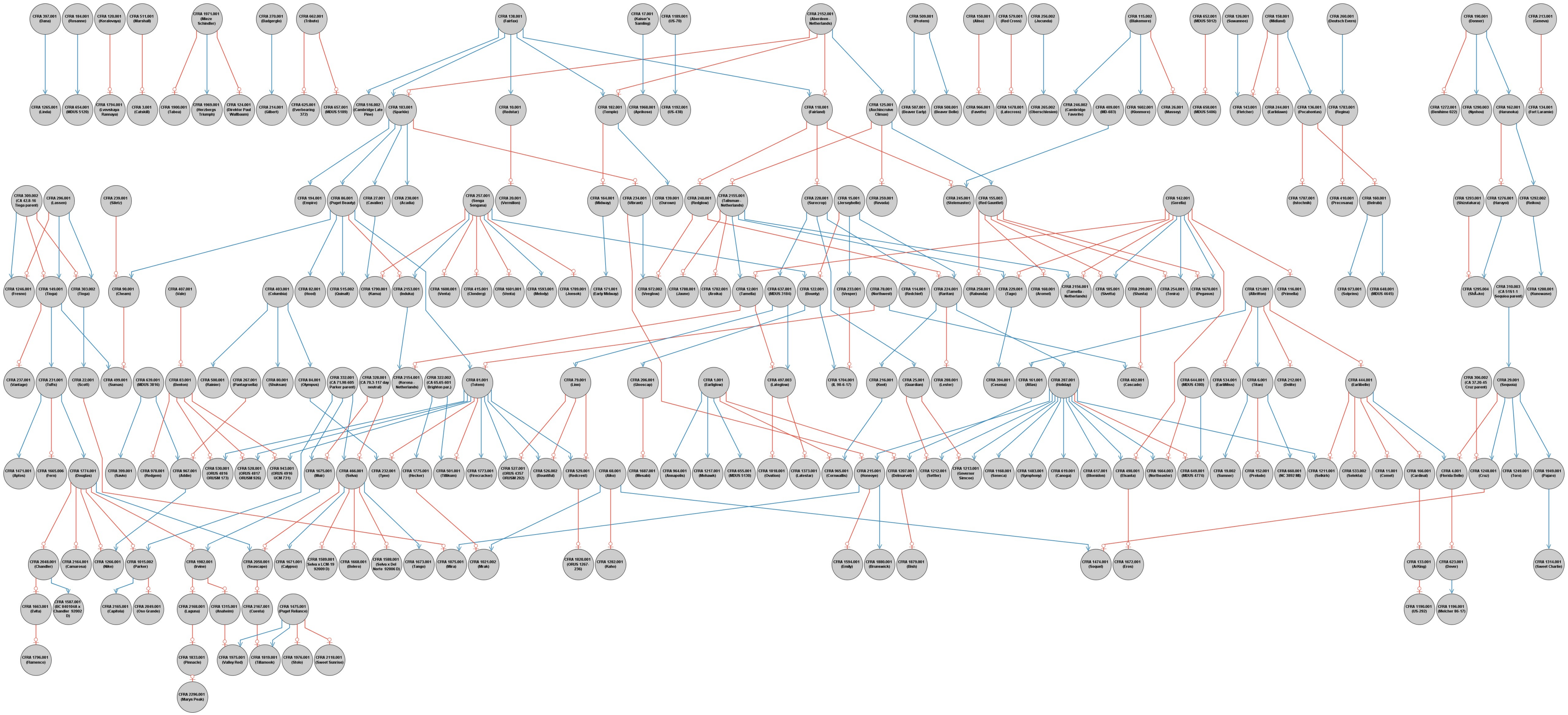

Supplement: Web_Material_uhac125 [file web_material_uhac125.zip › Supplementary_Fig_S2.pdf]
